# Stigmatizing Media Portrayal of Obesity During the Coronavirus (COVID-19) Pandemic

**DOI:** 10.3389/fpsyg.2020.02124

**Published:** 2020-09-03

**Authors:** Stuart W. Flint

**Affiliations:** ^1^School of Psychology, University of Leeds, Leeds, United Kingdom; ^2^Scaled Insights, Nexus, University of Leeds, Leeds, United Kingdom

**Keywords:** obesity, media portrayal, stigma, COVID-19, coronavirus

## Introduction

On 11th March 2020, the World Health Organization declared coronavirus disease 2019 (COVID-19) as a global pandemic (World Health Organisation, 2020). In response, countries around the world, identified several groups typically based on an underlying physical health condition (e.g., diabetes, COPD, HIV AIDS), older age and pregnancy status as at increased risk of severe illness and death (Australia: Australian Government Department of Health, 2020; Canada: Government of Canada, 2020; UK: Public Health England, 2020; USA: Centers of Disease Control Prevention, 2020). In the case of the USA and UK, a body mass index (BMI) of 40 kg.m^2^ or higher was included in this list, despite questions about why this was implemented as the cut-off point or whether this is an independent risk factor (Flint and Tahrani, 2020).

## Media Coverage of COVID-19 and Obesity

Media reports about COVID-19 and obesity have increased alongside the timeline of the pandemic and increasing number of deaths and ICU admissions reported for people with obesity—throughout which, a steady undertone of stigma toward people living with obesity has been evident. Indeed, media portrayal of obesity has long been identified as one of the most influential sources in contributing to the development and maintenance of weight stigma attitudes and discriminatory behavior (e.g., Saguy and Almeling, 2008; Hilbert and Ried, 2009; Heuer et al., 2011; Flint et al., 2016).

A recent example includes highly stigmatizing comments from Lord Robathan who criticized NHS Staff and teachers with overweight and obesity saying that “*They know what makes them fat and. dare I say it, they need to be shamed from eating and drinking too much*” (Wilcock, 2020). These comments are in stark contrast to the substantial evidence (e.g., Hayward et al., 2018) that demonstrates experiences of weight stigma and discrimination amongst people living with obesity are associated with maladaptive responses such as avoidance of healthcare, poorer eating behavior, and reduced physical activity. This coupled with reports that people are avoiding healthcare including not attending accident and emergency in hospitals or calling 999 (UK emergency number) due to a concern that they do not wish to “burden” the NHS is concerning (Campbell et al., 2020).

Framing of obesity as a societal “burden,” assigning blame and assigning individual responsibility, stigmatizing imagery, and language are well-known to contribute to the formation of stigmatizing attitudes and discriminatory behavior toward people living with obesity but also other health conditions. Media portrayal of obesity during COVID-19 has been no different. The impact of these media portrayals on influencing public opinion can be easily identified by reading the public responses sections or social media posts in response to media dissemination on these platforms.

Moreover, the potential impact of lockdown on reducing physical activity, greater snacking, and more consumption of foods high in saturated fat, sugar, and salt which has been a continual line of messaging in media, for instance, “*why lockdown is bad news for our kids' waistlines*” (Lally, 2020). Across the population, it is likely to influence the development of stigmatizing attitudes, and for many internalization of weight stigma that lead to unhealthy behavioral responses such as binge eating, reduced self-esteem, anxiety, and depression (e.g., Puhl et al., 2007; Täuber et al., 2018)—all of which can lead to further unintended consequences of COVID-19 lockdown that can be attributed to media portrayal and messaging.

Finally, it is imperative that given the increased anxiety amongst the general population during the pandemic, and for some groups—which may understandably include people identified as at increased risk of severe illness and death, that media take steps to assess the contributions of invited guests that might also lead to negative consequences. For instance, during the COVID-19 pandemic, Steve Miller, a hypnotherapist, appeared on UK television saying that “*if you are fat, you are on death row*,” “*the reason this country is getting fatter is because we suffer from ‘can't-be-bothered-itis'*,” that people living with obesity should “*take responsibility for their own health*,” that they should “*pay a premium for their healthcare*” and that they should take matters into their own hands and lose weight rather than “*blaming our genes*” (Sulway, 2020). These stigmatizing and simplistic perceptions are in stark contradiction to substantial evidence that demonstrates that obesity is a multifaceted, complex health condition (e.g., Butland et al., 2007). Indeed, in 2007, the UK Government commissioned Foresight project highlighted that that the “causes of obesity are extremely complex encompassing biology and behavior, but set within a cultural, environmental, and social framework” (Government Office for Science, 2007). Yet, despite this considerable and rapidly increasing empirical evidence of the complex, multifaceted nature of obesity, media portrayal has simplified the causes of obesity, blamed people living with obesity, and there has been little coverage of factors that are often outside of people's control.

## Conclusion

In sum, experiences of weight stigma and discrimination is known to negatively impact physical and mental health and during the COVID-19 pandemic, these experiences which may be either directly or indirectly result from media portrayal may have a significant impact on people living with obesity. In line with previous calls (e.g., Flint et al., 2018), it is thus, imperative that media portrayal is accurate with the evidence base, and both respectful and fair where efforts to ensure media content of obesity avoids unintended consequences through stigmatizing and discriminatory portrayal.

## Author Contributions

The author confirms being the sole contributor of this work and has approved it for publication.

## Conflict of Interest

The author declares that the research was conducted in the absence of any commercial or financial relationships that could be construed as a potential conflict of interest.
